# Phyllosphere microbes are associated with variety-specific accumulation of di-n-butyl phthalate in choysum (*Brassica parachinensis*)

**DOI:** 10.1128/aem.00958-25

**Published:** 2025-07-22

**Authors:** Rui-Ting Wu, Chang-Peng Ye, Qiong Hu, Yi-Min Gu, Huan Du, Miao-Yue Zhang, Lei Xiang, Bai-Lin Liu, Yan-Wen Li, Quan-Ying Cai, Ce-Hui Mo, Hai-Ming Zhao

**Affiliations:** 1 Guangdong Provincial Research Center for Environment Pollution Control and Remediation Materials, College of Life Science and Technology, Jinan University 506476 , Guangzhou, China; 2 Guangzhou Customs Technology Center 639043 , Guangzhou, China; 3 Guangdong Provincial Key Laboratory of Environmental Pollution Control and Remediation Technology, Sun Yat-Sen University 26469 , Guangzhou, China; The University of Tennessee Knoxville, Knoxville, Tennessee, USA

**Keywords:** phthalate esters, phyllosphere microbes, fumarate, low-accumulation variety, food safety

## Abstract

Plant-microbe interactions in the phyllosphere have been demonstrated to facilitate plant growth and enhance resistance to diverse stresses, but the resulting effect on bioaccumulation of contaminants in plants is rarely considered. Here, by comparing differences in the phyllosphere morphology, bacterial community assembly, and metabolites between two choysum (*Brassica parachinensis*) varieties differing in di-n-butyl phthalate (DBP) accumulation, we revealed associations between the key phyllosphere exudate (fumarate) and the recruited specific microbes that may contribute to the variety-specific DBP accumulation in choysum. Compared with the DBP-high accumulation variety (HAV), the DBP-low accumulation variety (LAV) could not only enhance the recruitment of phyllosphere microbes capable of degrading DBP by increasing fumarate secretion, but also facilitate the colonization of DBP-degrading bacteria via induction of biofilm formation, ultimately resulting in lower DBP accumulation in leaves. These findings offer novel insights into the LAV formation from the phyllosphere microbial perspective and highlight the role of phyllosphere microbes in mediating pollutant accumulation within crops, which is instrumental in minimizing pollutant accumulation through regulating the phyllosphere microbial community.

**IMPORTANCE:**

The bioaccumulation of phthalic acid esters (PAEs) in crops poses significant concerns for food safety, attracting considerable attention. Although existing studies have primarily elucidated the formation mechanisms of crop varieties with low PAE accumulation at both physiological and molecular levels, the role of phyllosphere microbiota remains uninvestigated. Specifically, the mechanisms through which these microbiotas mitigate PAE accumulation, along with the key exudate components involved, are still poorly comprehended. This study revealed the role of fumarate—a key phyllosphere exudate—and its recruited microbes in determining variety-specific PAE accumulation in choysum, based on the “cry for help” theory and supported by integrated microbiome and metabolome analysis. Furthermore, we provided direct evidence of how fumarate promoted the phyllosphere colonization of PAE-degrading bacteria and resulting reduction of PAE accumulation in plants. The novel findings highlight the crucial role of phyllosphere microbes in mediating pollutant accumulation within crops.

## INTRODUCTION

Due to the discharge of industrial and domestic wastes and the widespread utilization of plastic film, pesticides, and fertilizers, the contamination of agricultural soil by phthalic acid esters (PAEs) is increasingly exacerbated (1). The concentration of PAEs in Chinese agricultural soil exceeds the global average by a significant margin, reaching levels on the order of milligrams per kilogram (mg/kg) (2, 3). Consequently, the management of PAEs has emerged as a primary focus within the field of environmental endocrine disruptor management in China (4). The presence of PAEs in soil enables their absorption by roots and subsequent translocation to leaves (5, 6). Moreover, owing to their semi-volatile nature, PAEs can also volatilize into the atmosphere and permeate the cuticle wax on leaf surfaces, ultimately accumulating within the mesophyll tissue (6). Consequently, the concentrations of PAEs are usually found to be significantly higher in leaves compared to roots and rhizomes (7). The leaf constitutes the primary consumable component of vegetables, making ingestion of these agricultural products a prominent route for human exposure to PAEs, which poses a substantial threat to human health, ranging from birth defects and developmental disorders to severe conditions such as cancer and immunological dysfunctions (8, 9). Therefore, ensuring the secure production of agricultural products in the PAE-contaminated soils assumes paramount significance. Currently, the screening of low accumulating variety (LAV) of crops for the specific pollutant has emerged as a crucial strategy to ensure the safe production of agricultural commodities (10). The LAV of some crops for PAEs has been screened out, which exhibit acceptable low concentration of PAEs in comparison to their counterpart high accumulation variety (HAV) when cultivated in contaminated soil (11–17).

Exposure to PAEs induces ultrastructural damage to chloroplasts and inhibits photosynthetic electron transport chains, thereby disrupting photosynthetic systems and causing energy metabolism imbalance. Simultaneously, PAEs trigger reactive oxygen species (ROS) burst and dysregulation of antioxidant enzymes, leading to oxidative stress injury and collapse of the antioxidant defense system (12). It is found that the accumulation of PAEs is influenced by root morphology and cell wall structure, while the tolerance exhibited by plants toward PAEs is also associated with their accumulation, being facilitated by enhanced transpiration and tolerance mechanisms (7, 12). However, the plant microbiome also exerts an influence on the expression of plant traits in response to soil pollution. It is widely recognized that plants selectively recruit and shape rhizosphere microbiome based on their functional requirements, employing a well-known “cry for help” strategy (18, 19). By exudates, plants establish transboundary communication with resident microorganisms, forming a symbiotic relationship that harnesses their capabilities in pollutant degradation, plant growth promotion, and pathogen antagonism (20–22). Therefore, successful recruitment and maintenance of a sufficient abundance of specific microbial members is crucial to enhance the plant’s capacity to resist stresses (23–26).

In addition to the rhizosphere meaning the interface between roots and soil, the phyllosphere encompassing the leaf surface emerges as a pivotal component within the plant microbiome (27, 28). Phyllosphere microorganisms play a crucial role in mediating various ecological processes, such as facilitating plant growth through nitrogen fixation and hormone secretion, as well as exhibiting remarkable capabilities for stress response and pollutant degradation (29–31). The assembly of the phyllosphere microbial community exhibits a significant association with host defense, phosphate starvation response, cell wall integrity, ethylene signaling pathway, and the composition of phyllosphere exudates (32). Similar to the “cry for help” strategy observed in the plant rhizosphere, plants can also employ phyllosphere exudates to selectively recruit specific microorganisms to resist stresses (33). However, the role of phyllosphere microbes as a crucial factor in mediating bioaccumulation of contaminants within plants has been hardly investigated.

In this study, pot experiments were conducted with two choysum (*Brassica parachinensis*) varieties differing in di-n-butyl phthalate (DBP) accumulation (LAV and HAV). We hypothesize that there may be distinct microbial functional assemblies between the phyllosphere of two varieties, and that these variety-specific microbial community differences could potentially contribute to the observed variation in DBP accumulation. To achieve this, the phyllosphere bacterial community assemblies and leaf metabolite profiles of the two choysum varieties exposed to DBP were investigated using 16S rRNA high-throughput sequencing and non-targeted metabolomics methods. Subsequently, the key functional strains and key metabolites were identified, and their interactions were analyzed by chemotaxis assays and quantitative analyses. These results offer novel insights into the LAV formation from the phyllosphere microbial perspective and highlight the role of phyllosphere microbes in mediating pollutant accumulation within crops.

## RESULTS

### Effects of DBP exposure on the physiological characteristics and phyllosphere microbial colonization of choysum

**Fig 1:**
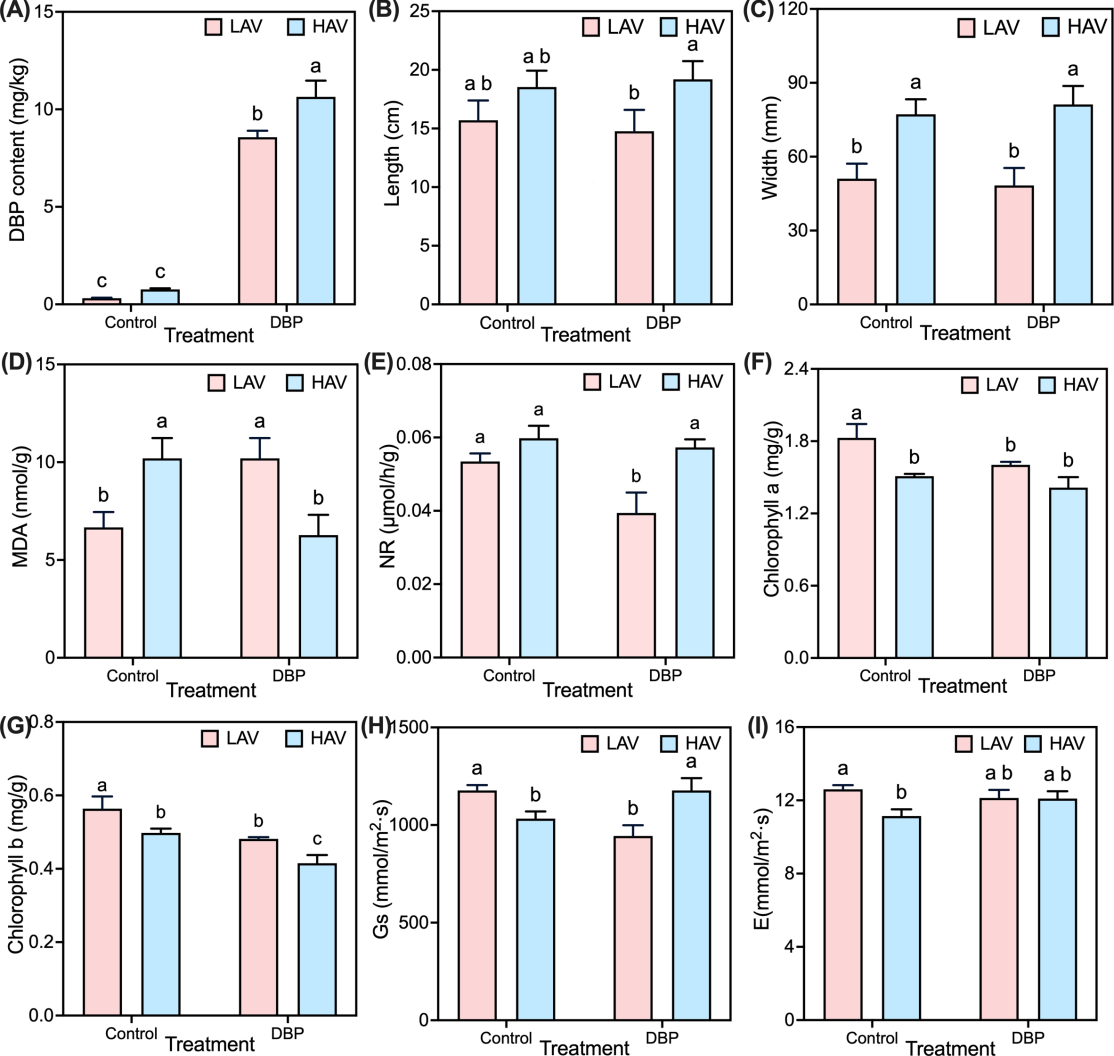
Leaf DBP accumulation (**A**), leaf length and width (**B and C**), enzyme activities of malondialdehyde (MDA) and nitrate reductase (NR) (**D and E**), content of chlorophyll a and b (**F and G**), and photosynthetic coefficients encompassing stomatal conductance (**H**) and transpiration rate (**I**) of the two varieties (HAV and LAV) under DBP treatments. Data are means ± SE (*n* = 6). The same lowercase letters represent no significant differences at the 0.05 level.

The DBP contents in the leaves were significantly (*P* < 0.05) lower in LAV than in HAV (Fig. 1A). The leaves of LAV were also significantly smaller in length and width (Fig. 1B and C). DBP exposure significantly increased DBP accumulation in both choysum varieties, with significantly higher DBP increase in HAV than in LAV (*P* < 0.05; Fig. 1A). Morphologically, DBP exposure led to no significant changes in leaf length and width for LAV and HAV between the controls and the DBP treatment groups but significant difference in leaf length in LAV than in HAV (*P* < 0.05), with significantly less leaf length and width in LAV than in HAV under DBP exposure (Fig. 1B and C). Physiological responses to DBP stress exhibited distinct patterns between the varieties. Under CK, malondialdehyde (MDA) content was significantly lower in LAV than in HAV, and nitrate reductase (NR) activity was comparable. However, MDA content was significantly higher in LAV than in HAV (*P* < 0.05), and NR activity was significantly lower (*P* < 0.05) in LAV than in HAV (Fig. 1D and E) under DBP stress. Photosynthetic parameters were generally reduced under DBP stress. These parameters, including chlorophyll (Chl a and Chl b), stomatal conductance (Gs), and transpiration rates (E) were all significantly higher in LAV than in HAV in the controls. However, the level difference in Chl a and E was comparable between LAV and HAV, while the Gs exhibited a significant reduction in LAV than in HAV under DBP exposure (Fig. 1G through I). Collectively, these results indicated that LAV suffered more severe DBP toxicity than HAV, exhibiting greater morphological alterations, stronger oxidative stress, and more pronounced photosynthetic impairment.

**Fig 2:**
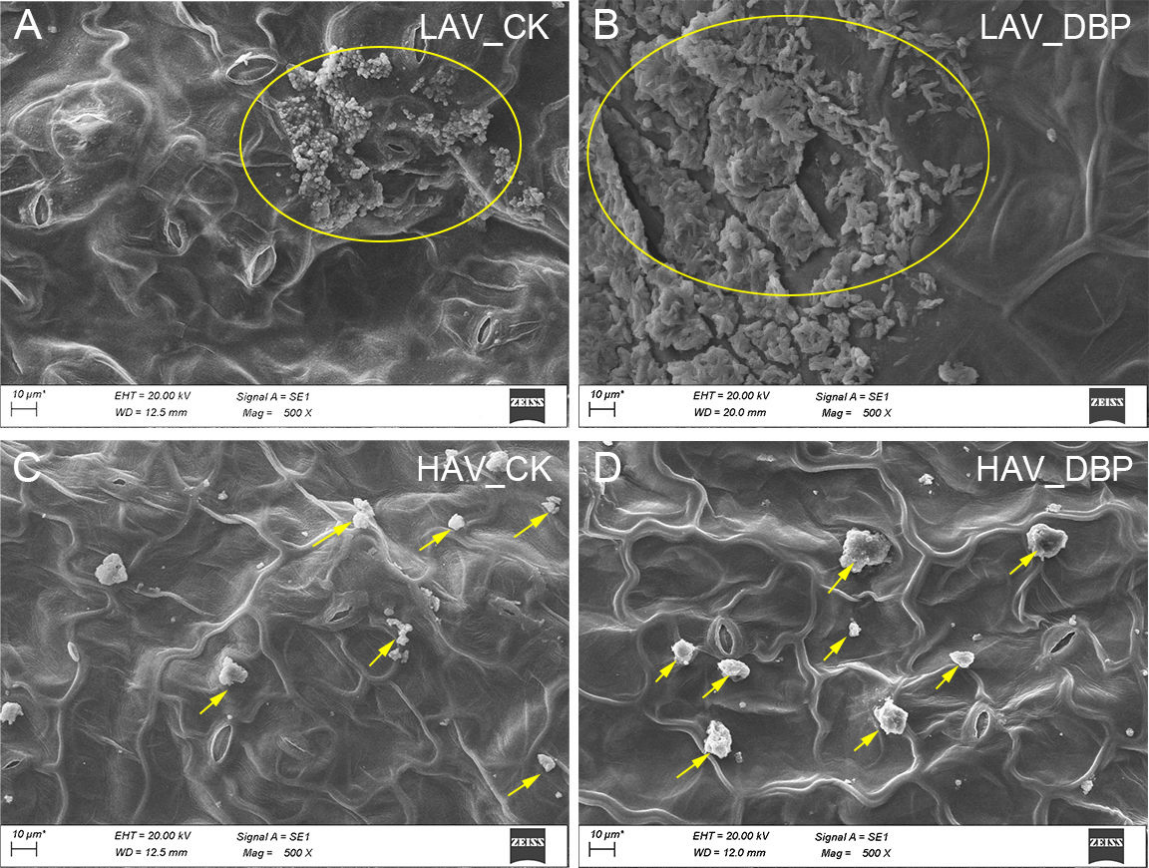
Scanning electron microscopy (SEM) analysis of microbial colonization on leaf surfaces of two choysum varieties (HAV and LAV) under DBP-contaminated and uncontaminated (CK) conditions. (**A and B**) Yellow circles refer to dense microbial aggregates on the leaf surface of LAV under uncontaminated and DBP-exposed conditions. (**C and D**) Yellow arrows refer to sparse microbial aggregates on the leaf surface of HAV under uncontaminated and DBP-exposed conditions.

Scanning electron microscope (SEM) analysis of leaf surfaces revealed distinct differences in phyllosphere microbial colonization between the two choysum varieties (Fig. 2). Notably, DBP exposure induced much more observable microbial aggregation and biofilm formation on LAV leaves than on HAV leaves. This divergence in spatial colonization patterns between the varieties likely corresponded to their differential DBP accumulation capacities.

### Alterations in phyllosphere microbial communities under DBP exposure

Exposure to DBP led to a reduction in βNTI (β nearest-taxon index) values in both LAV and HAV, suggesting a gradual transition from stochastic to deterministic process in the evolution of phyllosphere bacterial communities (Fig. S1A). The Chao1 index of phyllosphere microbes decreased significantly (*P* < 0.05) in HAV and insignificantly (*P* > 0.05) in LAV, implying a more pronounced disruption to the microbial community of HAV (Fig. S1B). Regarding the composition of the phyllosphere microbial community, exposure to DBP resulted in an increase in dominant phyla, which accounted for 90% of the community, and a decrease in rare phyla that constituted less than 5% of the community (Fig. S1C). Specifically, the relative abundance of the predominant phylum Cyanobacteria increased significantly (*P* < 0.05) in HAV and insignificantly in LAV (*P* > 0.05), with higher abundance than in LAV (Fig. S1D); and the relative abundance of the rare phyla Firmicutes and Bacteroidota in both varieties decreased significantly (Fig. S1E and F), with Firmicutes abundance considerably higher in LAV than in HAV (*P* < 0.05).

**Fig 3:**
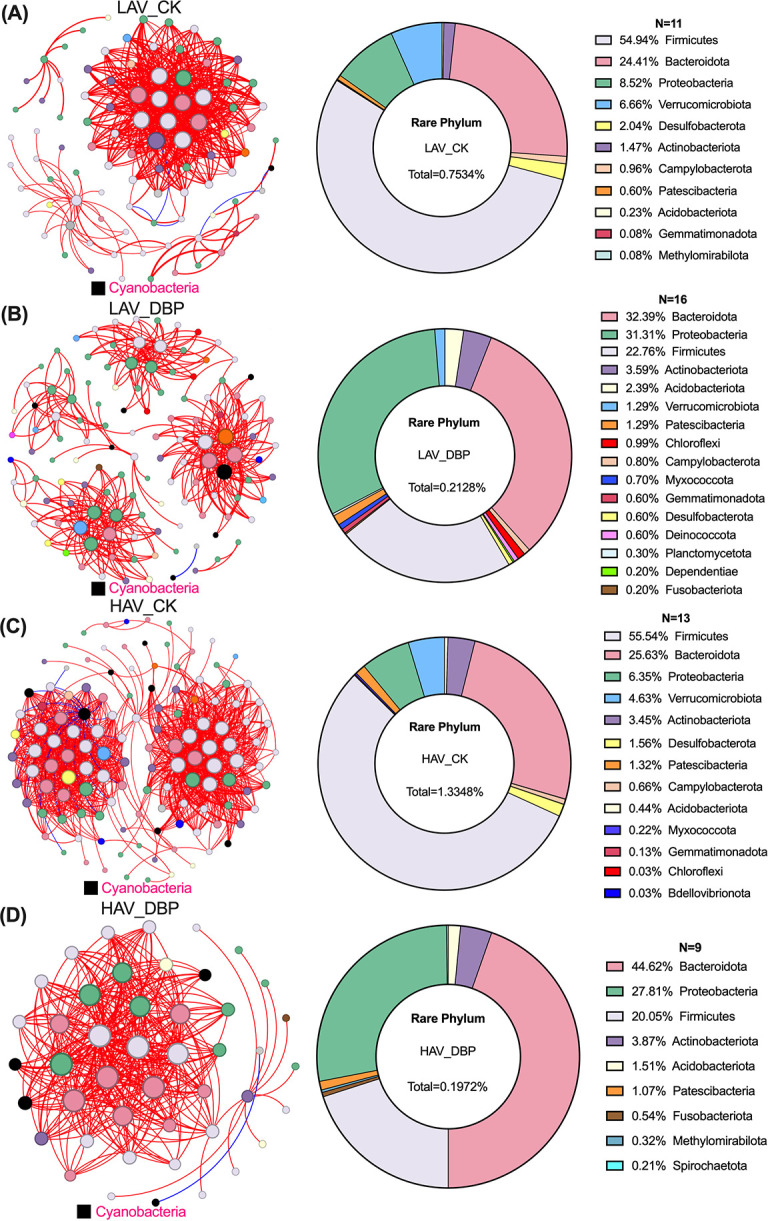
Co-occurrence networks of microbial communities and contribution rate of rare phylum of the LAV (**A and B**) and HAV (**C and D**) under DBP exposure.

DBP stress changed the community structure of phyllosphere microorganisms, increasing the number of rare phyla from 11 to 16 in LAV while decreasing it from 13 to 9 in HAV (Table S1; Fig. 3). Notably, low-abundance rare phyla (Firmicutes, Bacteroidota, and Proteobacteria) played a significant role in microbial community interactions within the phyllosphere. The relative abundance of Firmicutes declined to 22.76% in LAV and 20.05% in HAV, whereas Bacteroidota and Proteobacteria became the dominant phyla, surpassing Firmicutes with contributions of 33.39% and 31.31% in LAV and 44.62% and 27.81% in HAV, respectively (Fig. 3A through D).

### Effect of DBP exposure on leaf metabolites

**Fig 4:**
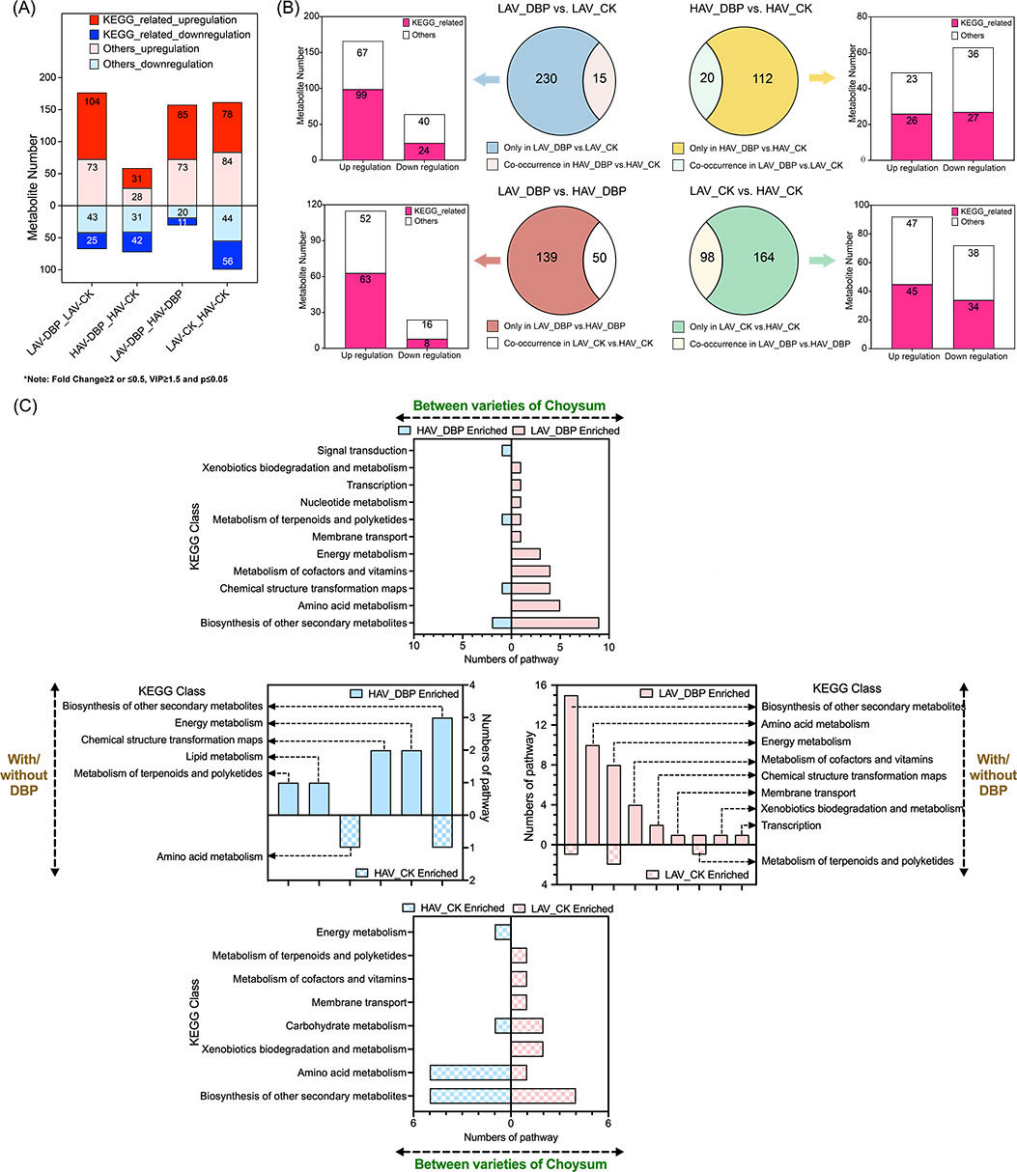
The number of differentially regulated metabolites associated with KEGG pathway by various comparative strategies (**A**). Differential metabolites encompass both co-occurred metabolites and sole metabolites identified by various comparative strategies, the number of differentially regulated co-occurred metabolites associated with KEGG pathway (**B**) and the classified number of KEGG enrichment (**C**).

Compared to the CK, exposure to DBP induced 177 (104 + 73) upregulated and 68 (43 + 25) downregulated differential metabolites (DMs) in LAV and 59 (31 + 28) and 73 (31 + 42) downregulated DMs in HAV. Pathway enrichment analysis demonstrated that LAV exhibited a greater number of KEGG-related upregulated DMs (85 vs 78) but fewer downregulated DMs (11 vs 56) related to HAV (Fig. 4A). Following removal of co-occurring interfering metabolites, DBP exposure caused more pronounced metabolic alterations in LAV than in HAV (230 vs 112). Among these, significantly more KEGG-related upregulated DMs occurred in LAV than in HAV (99 vs 26). Notably, up to approximately eightfold higher increase in upregulated DMs was observed in LAV than in HAV (63 vs 8) (Fig. 4B). These results collectively indicate that DBP exposure induces more pronounced upregulation of metabolic pathways in LAV than in HAV.

**Fig 5:**
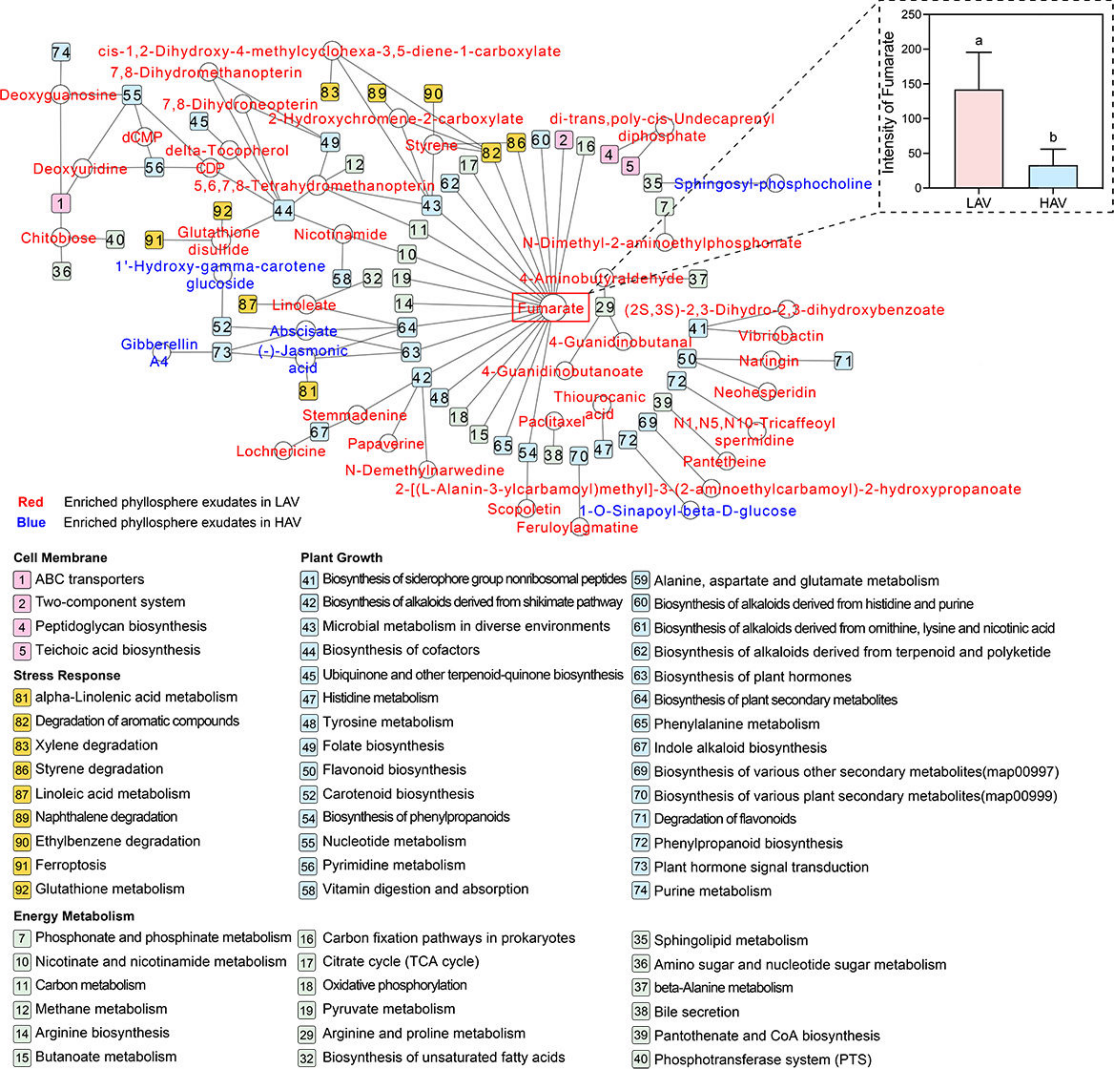
Key phyllosphere metabolites involved in KEGG pathways of the two varieties under DBP exposure. The inserted bar chart represents the peak intensity of fumarate between different varieties exposed to DBP. Data are means ± SE (*n* = 6). The same lowercase letters represent no significant differences at the 0.05 level.

Further enrichment and classification analysis of metabolic pathways demonstrated varietal differences in response to DBP stress (Fig. 4C). Upon exposure to DBP, HAV exhibited diminished amino acid metabolism but elevated expression in signal transduction pathways, indicating relatively minor metabolic disruption in its phyllosphere. Compared to HAV, LAV exhibited a more pronounced upregulation of xenobiotics degradation following DBP exposure, suggesting the colonization of degradative microorganisms in the phyllosphere. According to the enrichment network (Fig. 5), fumarate exhibited a fourfold higher increase in LAV leaves compared to HAV leaves following DBP exposure (*P* < 0.05).

### Role of fumarate in colonization of functional microorganisms

**Fig 6:**
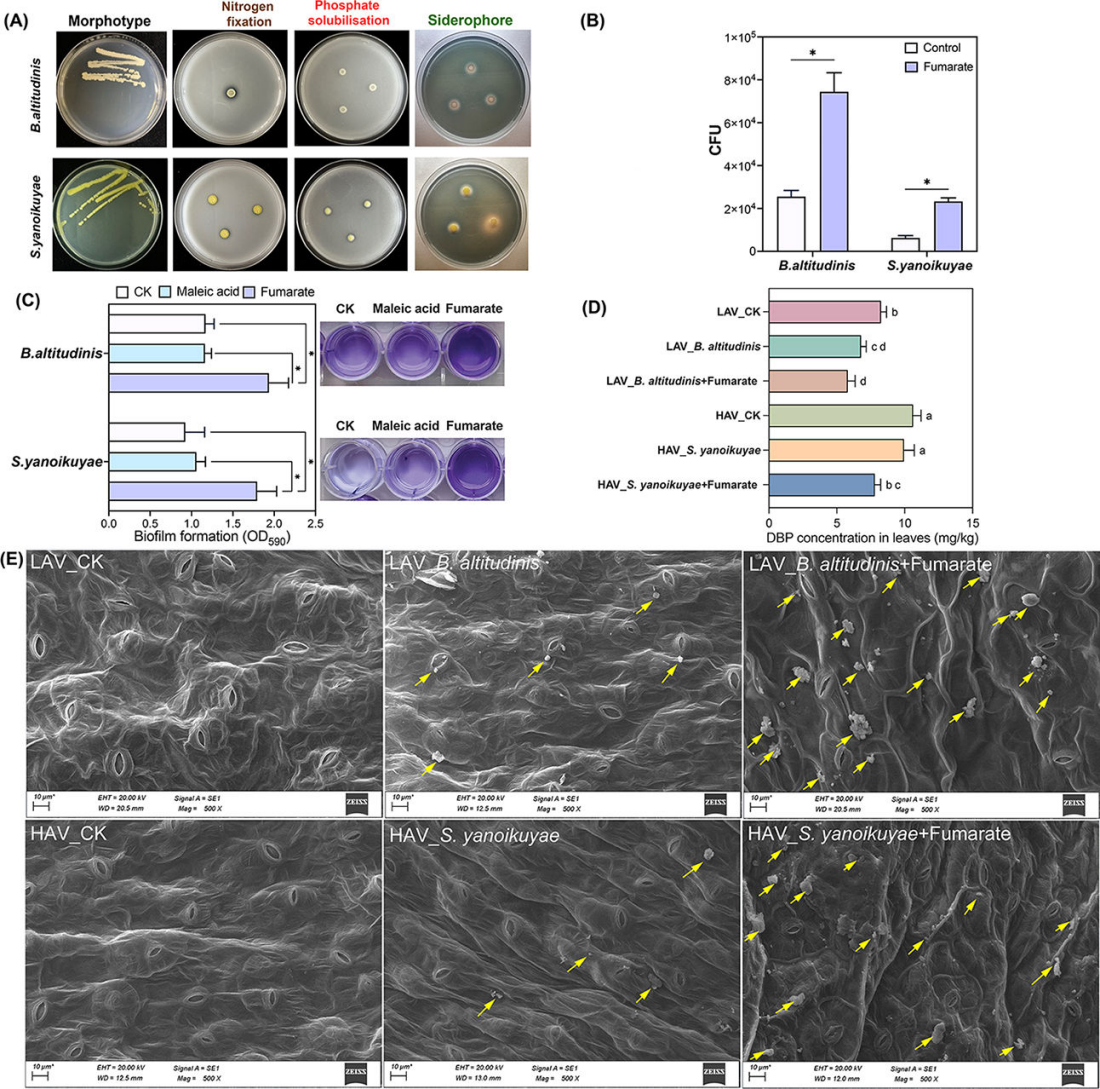
(**A**) Characterization of colony morphotype, nitrogen fixation, phosphate solubilization, and siderophore production of the two isolated bacterial strains. Corresponding quantitative measurements are provided in Table S2. (**B**) Colony-forming unit (CFU) counting of the two strains based on the capillary assay of the two strains toward fumarate (100 µmol/L), maleic acid (100 µmol/L), and dd H_2_O (control) evaluated by capillary assay. Bars indicate the standard errors of the means from three replicates. Columns marked with an asterisk (*) indicate statistically significant differences as determined by Duncan’s test (*P* < 0.05, hereinafter inclusive). (**C**) Biofilm formation of the two strains induced by fumarate (100 µmol/L), dd H_2_O (negative control), and maleic acid (positive control). Biofilm indexes as quantified by crystal violet staining (OD_590_) from at least three biological replicate cultures with four technical replicates each. (**D**) DBP accumulation in leaves of the two choysum varieties under different inoculation treatments. The same lowercase letters represent no significant differences at the 0.05 level. (**E**) SEM images of microbial colonization on the leaf surfaces of two choysum varieties under different inoculation treatments. Yellow arrows refer to microbial aggregates.

Given the significantly lower accumulation of DBP in LAV compared to HAV, along with its substantially higher fumarate secretion, we infer that fumarate regulates the colonization of DBP-degrading microorganisms, thereby modulating DBP accumulation in leaves. The hypothesis was confirmed by the isolation of two strains, *Bacillus altitudinis* from LAV and *Sphingobium yanoikuyae* from HAV, which exhibited both DBP degradation capability and plant growth-promotion activity (Fig. 6A; Table S2). Chemotactic assays demonstrated positive attraction of DBP-degrading microorganisms toward 100 µmol/L fumarate (Fig. 6B), highlighting the pivotal role of fumarate in facilitating their colonization. Biofilm formation assays also showed that 100 µmol/L fumarate significantly enhanced the biofilm formation of phyllosphere isolates (Fig. 6C).

Furthermore, the vital role of fumarate in enhancing phyllosphere colonization of DBP-degrading bacteria and subsequently reducing DBP accumulation in leaves was corroborated by the foliar inoculation experiment. As shown in Fig. 6D, foliar co-application of bacteria and fumarate significantly (*P* < 0.05) reduced DBP levels in both choysum varieties compared to the blank control or bacterial inoculation alone. Consistently, SEM imaging (Fig. 6E) revealed that fumarate supplementation markedly improved colonization of *B. altitudinis* and *S. yanoikuyae* on leaf surfaces of both varieties. Moreover, standalone bacterial inoculation showed that LAV facilitated greater colonization than HAV, likely due to its higher self-secreted fumarate, which may confer a competitive advantage in phyllosphere establishment. Overall, we can conclude that LAV enhances fumarate secretion to recruit more DBP-degrading microorganisms on the phyllosphere, consequently resulting in less DBP accumulation in leaves when compared with HAV.

## DISCUSSION 

### DBP facilitates the formation of microbial aggregate on leaf surface of LAV

Similar to our previous result (12), LAV exhibited significantly lower DBP concentrations in leaves relative to HAV under DBP treatment (Fig. 1A). Morphometric analysis revealed substantially smaller dimensions in LAV related to HAV (Fig. 1B and C). Current understanding suggests that phyllosphere architecture critically governs microbial community assembly and contaminant sequestration, potentially explaining the reduced DBP accumulation in LAV (34, 35). The production of MDA was indicative of peroxidase-catalase level in the antioxidant defense system, which exerted a dominant influence on the community structure of phyllosphere microorganisms (36, 37). The expression of NR was not only related to the capacity to accumulate pollutant, but also correlated with the diversity of phyllosphere microorganisms (37–39). Comparative analysis revealed that LAV exhibited greater oxidative stress susceptibility (elevated MDA) and impaired nitrogen assimilation (reduced NR activity) relative to HAV (Fig. 1D and E). Therefore, the weaker antioxidant capacity of LAV was found to be associated with its lower accumulation of DBP and the heightened microbial aggregate on leaf surface.

As a crucial indicator of internal metabolism, any inhibition in photosynthesis can lead to detrimental effects on physiological activities during plant growth (40). The inhibition of DBP on the synthesis of chlorophyll precursor substances resulted in the destruction of chloroplast structure and a decrease in chlorophyll content (Fig. 1F and G), ultimately leading to reduced photosynthetic efficiency (12, 41). Moreover, the photosynthetic efficiency is found to be associated with gas exchange parameters, suggesting that leaf-air gas exchange partially regulates the accumulation of various semivolatile organic compounds by plants (12). In this study, the lower Gs observed in LAV indicated a reduced stomatal resistance, which directly influenced the diffusion of DBP through the stomata (Fig. 1H), potentially resulting in a decreased accumulation of DBP in LAV via the stomata. The exposure to DBP resulted in a reduction of transpiration rates in LAV (Fig. 1I). Previous studies have demonstrated a positive correlation between transpiration rates and the translocation rates of DBPs in plants, as reduced transpiration may hinder the root-mediated transport of pollutants (12, 41). Hence, the reduction in transpiration resulted in a decreased accumulation of DBP in the leaves of LAV. The presence of DBP affects water transport to the shoot by inhibiting transpiration, as evidenced by the reduced leaf size and water potential, which may potentially cause water deficit in the leaves of LAV (42, 43). While bacterial aggregates can enhance leaf wettability by producing surfactants and hygroscopic extracellular polysaccharides (44). Consequently, a reduction of transpiration rates in LAV resulted in smaller leaf dimensions and enhanced colonization of phyllosphere microorganisms on the leaf surface.

Most microorganisms on leaf surfaces exist as large aggregates, embedded within extracellular polymeric substances (EPS) (45). EPS production is not only associated with oxidative stress damage but also contributes to the release of catalase (46). Consequently, due to a higher degree of severe oxidative damage in LAV leaves induced by DBP, the leaf surface becomes more susceptible to EPS generation and aggregate formation compared to HAV leaves (Fig. 2). As a structural framework facilitating the formation of biofilm architectures, EPS offer a 3D matrix for microbial communities to establish biofilms, thereby enabling efficient adaptation of bacterial masses to be challenging environmental conditions (47). Previous studies have demonstrated that PAEs can induce biofilm formation, highlighting the significant advantages of bacterial biofilms in environmental and agricultural applications, particularly for organic pollutant degradation (48). Hence, the observed biofilm on leaf surface of LAV may be closely associated with its distinctive low-DBP accumulation characteristics.

### DBP affects the assembly of phyllosphere microorganisms

Recent studies have established a relationship between stochastic/deterministic processes and microorganisms, demonstrating that environmental disturbance facilitates a shift in their balance (49). Exposure to DBP exerted selective pressure on phyllosphere microbial communities, driving deterministic assembly processes that led to distinct bacterial community structures (Fig. S1A). This effect was more pronounced in LAV than in HAV, likely due to LAV’s selective recruitment of biofilm-formation bacteria under DBP stress, which contributed to reduced accumulation of DBP (Fig. 2). Consequently, deterministic selection predominated over stochastic processes during this disturbance event (50). Overall, our results demonstrated that DBP-exposed phyllosphere microbial communities exhibited varietal differences in the balance between stochastic and deterministic assembly processes. The varietal specificity in community assembly directly correlated with differential DBP accumulation patterns between LAV and HAV (51).

In accordance with previous findings (52), the presence of DBP significantly impacted the composition of the phyllosphere microbial community and resulted in a reduction in microbial community diversity index (Fig. S1B). Rare bacteria (Firmicutes and Bacteroidota), though constituting less than 2% of the community, exhibited significant abundance reductions in both varieties’ phyllosphere microbiota (Fig. S1E and F). Notably, these rare genera became the key mediators of DBP-induced community interactions (Fig. 3). Given the established role of rare bacteria in maintaining ecological stability and community functionality (53), their observed population decline directly contributed to the reduced diversity in phyllosphere microbial communities.

Apart from plant and environmental factors, the assembly processes of microbial communities were also influenced by microbial interactions (51). In this study, the phyllosphere microbial community networks of both varieties exposed to DBP demonstrated a nonrandom pattern, indicating that the deterministic processes imposed by DBP stress played a more significant role in microbial interactions. DBP exposure reduced microbial network complexity in both varieties, likely resulting from diminished species coexistence and functional redundancy within bacterial communities. This suggests that biological interactions play a crucial role in shaping the assembly of bacterial communities (54). Notably, rare bacterial genera from Firmicutes, Bacteroidota, and Proteobacteria, known to contribute to PAE degradation (22, 55), showed particularly significant responses to DBP exposure. On the LAV leaf surface, the relative abundance of Firmicutes significantly increased, whereas it decreased on the HAV (Fig. S1E). This differential response suggests that colonization of PAEs-degrading bacteria may be associated with the variety-specific accumulation of DBP in choysum. Notably, Cyanobacteria remained the dominant phylum in the phyllosphere, consistent with previous findings (56). DBP exposure significantly increased the relative abundance of Cyanobacteria in HAV phyllosphere (Fig. S1D), exerting a greater influence on the community interaction network. Given this phylum’s known growth-promoting functions (57), these abundance changes may contribute to the observed leaf dimension increases in HAV.

### Phyllosphere colonization of degrading microorganisms mitigates DBP accumulation in leaves

Elevated oxidative stress induced by DBP exposure resulted in a greater disturbance of foliar metabolite profiles in LAV compared to HAV (Fig. 4A and B). The visual analysis of KEGG enrichment suggested that DBP exposure resulted in significant disturbance of plant basic metabolic pathways in LAV, such as amino acid metabolism, energy metabolism, and metabolism of cofactors and vitamins (Fig. 4C), which could put the leaf metabolism out of homeostasis. Consequently, LAV leaves under DBP stress might actively seek help from the phyllosphere microbes mentioned above (58). By contrast, HAV leaves demonstrated enhanced expression in both signal transduction pathways and terpenoids/polyketides metabolism, indicating greater adaptive capacity to mediate abiotic stress responses (59, 60). The upregulation of these pathways could simultaneously promote plant growth and activate plant defense responses, ultimately enhancing antioxidant capacity and maintaining metabolite homeostasis in HAV leaves (Fig. 4B).

**Fig 7:**
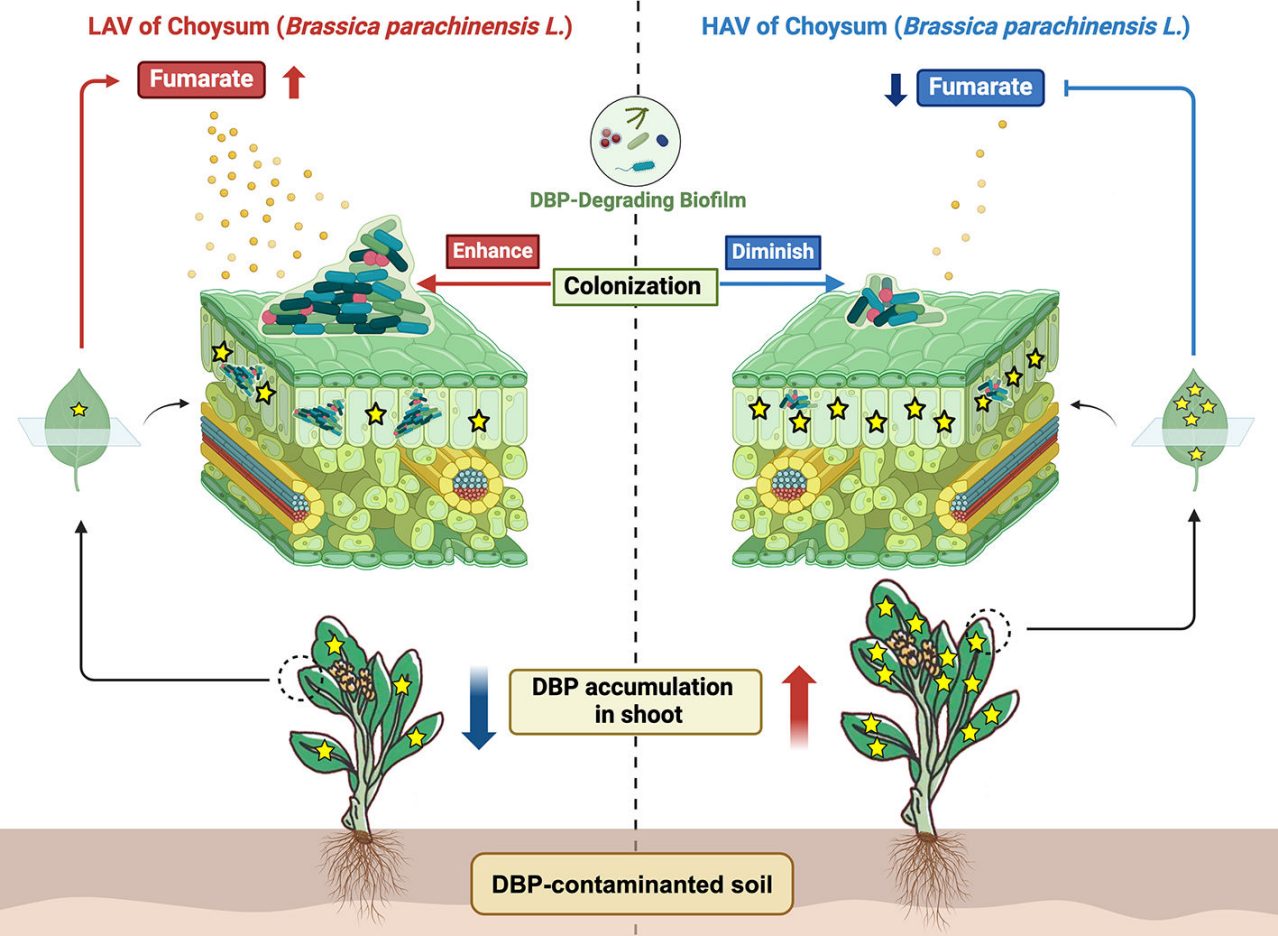
Schematic representation of the mechanism underlying the effect of fumarate on the phyllosphere microbe recruitment driven by biofilm formation and subsequent accumulation of DBP in choysum. The redness and blueness of the lines denote promotion and inhibition, respectively.

Numerous studies have revealed the role of exudates (mainly root exudates) in mediating plant-microbe interactions and abiotic stress adaptation (61). Our previous study specifically identified low-molecular-weight organic acids as key regulators of ciprofloxacin accumulation in choysum (*B. parachinensis*) (24). In this study, we revealed the pivotal roles of fumarate, a key phyllosphere exudate, in determining the variety-specific accumulation of DBP in choysum by recruiting DBP-degrading bacteria. A compendious mechanism model (Fig. 7) is proposed: under DBP exposure, the LAV leaves enhance fumarate secretion, promoting the colonization of DBP-degrading bacteria by inducing biofilm formation, which reduces DBP accumulation in leaves. In comparison, the HAV leaves secrete less fumarate, limiting the colonization of DBP-degrading bacteria and thus increasing DBP accumulation. This suggests that LAV strategically increases fumarate secretion to recruit DBP-degrading bacteria in the phyllosphere, thereby mitigating DBP accumulation in its leaves. These findings suggest a potential association between phyllosphere microbes and DBP accumulation, but further experimental validation is required to establish causality.

Overall, our findings emphasize the pivotal roles of phyllosphere microorganisms in maneuvering DBP accumulation in crops. Fumarate facilitates the colonization of degrading microorganisms by inducing biofilm formation, while its secretion levels exhibit significant variation between the two choysum varieties. This differential secretion pattern, to a considerable extent, determines the cultivar-specific DBP accumulation in leaves and their adaptive capacity to DBP exposure. To the best of our knowledge, this is the first report on the secretion of phyllosphere exudates in response to pollution stress and its significant impact on the phyllosphere microbial colonization and the consequence DBP accumulation. These results provide new insights into the role of phyllosphere microorganisms in adjusting pollutant accumulation in crops, which can be harnessed to minimize contamination by applying key phyllospheric exudates and/or functional microorganisms to the leaves.

## MATERIALS AND METHODS

### Chemicals and plant materials

DBP (98.7% purity) and its standard solution (1,000 µg/mL in dichloromethane, 99.8%) were procured from Sigma Chemical Co., USA. Other chemicals were sourced from Tianjin Chemical Reagent Co., China. Seeds of the two choysum varieties, namely LAV (Lvbao) and HAV (Huaguan), were purchased from the Vegetable Research Institute, Guangdong Academy of Agricultural Science, China.

### Soil preparation and experimental design

The soil for pot experiments was collected from an experimental field (depth: 0–20 cm layer) at Guangzhou, China. The physicochemical properties of soil and the preparation of DBP-contaminated soil are presented in supplementary Text S1. Seeds of the two choysum varieties were surface-sterilized with 75% ethanol solution for 30 s, followed by immersion in 2% sodium hypochlorite solution for 10 min. After germinating in a sterile vermiculite for 14 days, the seedlings of two varieties were subsequently transplanted into pots containing 6 kg of contaminated soil. Concurrently, plant grown in uncontaminated soil was established as control groups. In total, four treatments were implemented, with each treatment being replicated six times. The plants were randomly arranged in a glass-enclosed greenhouse, ensuring the preservation of natural day-night conditions, maintaining a temperature ranging from 25°C to 32°C and a relative humidity of 60%. Replenishment of deionized water was conducted every alternate day to maintain appropriate soil moisture content. After 45 days of cultivation, the uniform and healthy leaves were sampled using sterile scissors, and each sample yielded approximately 60 g of leaves.

### Determination of DBP accumulation in leaves

The procedures for ultrasonic-assisted sample extraction using a silica gel column and DBP analysis via gas chromatography coupled with mass spectrometry (GC/MS, Shimadzu QP2010 Plus, Japan) were conducted according to the method described in our previous study with minor modifications (see supplementary Text S2) (12). The limit of detection for DBP was determined at 1.2 µg/kg. In all procedural blanks (*n* = 12), the average concentration of DBP was 3.2 µg/kg, with a range from 1.27 to 7.80 µg/kg, which was subtracted from the sample values accordingly. The recovery rates of DBP in plant samples ranged from 87.4% to 107.2%.

### Physiological measurements of leaves

The length and width of the fresh leaves were measured by electronic vernier caliper. The fresh leaves (1.0 g) were weighed and then added into the liquid nitrogen to grind. The resulting homogenate was mixed with PBS, centrifuged at 2°C and 8000 r/min for 10 min. The supernatant was collected for determination of MDA and NR activity followed by kits (Nanjing Jiancheng Bioengineering Co., Ltd., China) (12, 62). The extraction of chlorophyll a (chl a) and chlorophyll b (chl b) from 0.2 g of fresh leaves was performed with acetone-ethanol solution (63). Subsequently, the quantification of two chlorophylls was measured by ultraviolet spectrophotometer with their absorbance at 663 and 645 nm, respectively. The contents of two chlorophylls were determined by the following formulas:


$$Chl\;\text{a}\;\text{(}\text{mg/}\text{g}\text{)}\;\text{=}\frac{\text{(}\text{12.71}A_{663}-\text{2.59}A_{645}\text{)}\;\times \;V}{\text{1,000FW}}$$

$$Chl\;\text{b}\;\text{(}\text{mg/g}\text{)}\;\text{=}\frac{\text{(}\text{22.88}A_{\text{645}}-\text{4.67}A_{\text{663}}\text{)}\;\times \;V}{\text{1,000FW}}$$

where *V* represents the total volume of the extract (in mL); FW denotes the fresh weight of leaves (in g); and *A*_663_ and *A*_645_ correspond to absorbance values.

The photosynthetic efficiency analyzer CIRAS-3 (PP Systems, USA) was used to determine the photosynthetic coefficient of choysum before destructive sampling (63). During the measurement, the temperature of the leaf chamber was set at 28°C, and natural gas supply from the buffer bottle ensured a stabilized CO_2_ concentration of 460 µmol/ mol within the chamber. The flow rate was maintained at 300 µmol/s, while a single point received an irradiance of 1,000 µmol/(m^2^·s^−1^). Stomatal conductance (Gs) and transpiration coefficient (E) were subsequently measured. The measurements for each treatment were repeated 15 times.

### Phyllosphere microbiome amplicon analysis

The leaves (10.0 g) were finely immersed in a phosphate-buffered solution (PBS) containing the surfactant Silwet L-77 (0.01 M, pH 7.4) (64). Subsequently, the suspension was shaken at 200 r/min at room temperature (25°C) for 2 h. One proportion of collected suspension was utilized for total DNA extraction following the instruction manual of PowerSoil Isolation Kit (MoBio Laboratory, CA, USA). The integrity and purity of DNA were assessed by 1% agarose gel electrophoresis, and the concentration of DNA was checked by Qubit 3.0 fluorometer (Thermo Fisher Scientific, MA, USA). The V3–V4 hypervariable region was PCR-amplified using the primers 338F (5′-ACTCCTACGGGAGGCAGCA-3′) and 806R (5′-GGACTACHVGGGTWTCTAAT-3). PCR amplification, sequencing construction, and sequencing were performed by Beijing Biomarker Technologies Co., Ltd. (Beijing, China). The obtained library was constructed using NEBNext Ultra DNA Library Prep Kit (New England Biolabs, MA, USA) on the Illumina NovaSeq 6000 platform (Illumina, USA). The raw image data files obtained from sequencing were converted into raw reads and the results were stored in FASTQ format. The raw reads were uploaded into QIIME2 (version 2020.6) for quantitative insights (65). Subsequently, the clean data underwent filtering using FLASH, Trimmomatic (version 0.33), and Cutadapt (version 1.9.1) (66). The assembled reads were assigned to amplicon sequence variants (ASVs) using USEARCH (version 10) in conjunction with the UCHIME algorithm (version 8.1), employing a 97% cutoff (67, 68).

### Metabolomics analysis of phyllosphere exudates

The fresh leaves (10 g) were immediately immersed in liquid nitrogen for grinding, and the metabolites were analyzed following previously established protocols (69). The LC-MS/MS analyzes were performed using an Acquity I-Class PLUS ultra-high performance liquid (Waters, USA) coupled to an Xevo G2-XS QTof high resolution mass spectrometer (Waters), equipped with a Waters Acquity UPLC HSS T3 column (2.1 mm × 100 mm, 1.8 µm). The mobile phase consisted of 0.1% formic acid aqueous solution (solvent A) and 0.1% formic acid acetonitrile (solvent B). The parameters of the electrospray ionization ion source were as follows: capillary voltage: 2,000 V (positive ion modes) and −2,000 V (negative ion modes); cone voltage: 30 V; ion source temperature: 150°C; desolvent gas temperature: 500°C; backflush gas flow rate: 50 L/h; and desolvent gas flow rate: 800 L/h.

The mass spectrometry data collected using MassLynx V4.2 (Waters) were processed by Progenesis QI software for peak extraction, alignment, and normalization. Then, the METLIN database was applied in metabolite identification. The significantly different metabolites were identified using an OPLS-DA model and *t*-test based on the following screening criteria: a VIP value > 1.5 and a *P* value < 0.05, along with a fold change (FC) greater than 2 or less than 0.5. The significantly different metabolites were annotated and classified by KEGG databases, and the network of KEGG enrichment was reconstructed by Cytoscape (version 3.10.2).

### Morphotype of leaf surface measured by SEM

The fresh leaves were cut into 5 mm × 5 mm fragments and immediately immersed in a 10 mL 2.5% glutaraldehyde, standing at 4°C overnight. Subsequently, the leaves underwent sequential treatments with 30%, 50%, 60%, 70%, 80%, 90%, 95%, and 100% ethanol solutions for a duration of 15 min per treatment. Following dehydration, the leaves were dried using CO_2_ critical point drying for 2 h (70). The leaf surface morphology was observed by SEM (EVO MA 15, ZEISS, Germany) equipped with secondary electron, an extra high tension of 20 kV and a magnification (Mag) of 500×.

### Degrading bacteria isolation and function identification

Leaf samples from two choysum varieties were aseptically collected and individually stored in sterile bags. For microbial suspension preparation, 1 g aliquots of leaf tissue was homogenized in 9 mL of sterile water followed by 60 min of continuous agitation. The gradient acclimation method was employed to isolate efficient DBP-degrading bacterial strains (22). A sequential acclimation protocol was implemented using DBP concentrations of 100, 200, 400, 600, 800, and 1,000 mg/L, with each acclimation phase maintained for 7 days. For bacterial isolation, 100 µL aliquots of the acclimated cultures were plated in triplicate onto mineral salt medium agar plates containing 100 mg/L DBP as the sole carbon source. After 72 h of incubation at 28°C, distinct colonies were carefully isolated for subsequent DBP-degradation capability assessment. From leaves of each choysum variety, a bacterial isolate demonstrating optimal DBP-degradation performance was selectively identified as a potential candidate strain. The candidate strains were identified through 16S rRNA gene sequencing (71), and their plant growth-promoting traits, including nitrogen fixation, phosphate solubilization, and siderophore production, were evaluated according to established methodologies (72).

### Chemotaxis and biofilm formation assays

The modified capillary assay was conducted following the previously established methodology to quantitatively assess the chemotactic response of the functional strains toward fumarate (73). The functional strains were cultivated in LB media until an OD_600_ of 1.0 was attained. Subsequently, the cells were harvested by centrifugation and then washed twice with the aforementioned chemotaxis buffer and resuspended in the same buffer (OD_600_ = 1.0). 10 mL of cell suspension prepared above was added to a Petri dish 60 mm. Standard 1  µL capillaries loaded with 100  µL concentrated fumarate (100 µmol/L) and maleic acid (100 µmol/L) were immersed in the cell suspension, while the chemotaxis buffer (dd H_2_O) was performed as the negative control. After incubating at room temperature for 45  min, the liquid from each capillary was transferred into sterilized tubes. The suspension was subsequently diluted to 10^−3^, 10^−4^, and 10^−5^ and plated on LB plates. The colony-forming unit (CFU) was determined by incubating the plates at 30°C for 24  h. Each treatment was replicated three times.

Each well of a 24-well plate was supplemented with 1 mL LB broth, half of which contained 100 µmol/L fumarate. Following the addition of bacteria at a volume ratio of 0.1% (vol/vol), the broth was incubated at 30°C for 48 h. Biofilm formation was assessed by crystal violet staining and enzyme-labeled instrumentation, with wavelength measurements at OD_590_ (74).

### Foliar inoculation experiment

Two-week seedlings of two choysum varieties (LAV and HAV) were transplanted into pots containing sterile soil contaminated with 100 mg/kg DBP. Bacterial suspensions of two isolates (*B. altitudinis* and *S. yanoikuyae*) were prepared in PBS at 1.0 × 10⁸ CFU/mL, respectively. To evaluate the role of fumarate in promoting microbial colonization, three treatments were applied per choysum variety: (i) Control (PBS spray); (ii) Microbial inoculation (LAV_*B. altitudinis*, HAV_*S. yanoikuyae*); (iii) Microbial inoculation + fumarate (LAV_*B. altitudinis* + Fumarate, HAV_*S. yanoikuyae +* Fumarate). Bacterial suspensions were applied as a single foliar spray (until runoff) on Day 1 (75). Fumarate (100 µmol/L) was subsequently sprayed until runoff occurred on both leaf surfaces at 7-day intervals (Days 1, 8, 15, 22, and 29) (76). PBS-treated plants served as controls, with three replicates per treatment. The pot experiment was conducted in a sterile glass greenhouse. After 30 days of growth, leaves were harvested to assess microbial colonization and DBP accumulation.

### Statistical analysis and visualization

Statistical analyses were conducted using Prism 10 (version 10.2.2) and R software (v4.3.3, https://www.r-project.org/). Community alpha diversity indexes based on 16S rRNA amplicon sequencing data were calculated with the “vegan” package. The beta diversity was analyzed using principal coordinates analysis based on Bray-Curtis dissimilarity. Differences in the relative abundance of ASVs between treatments were detected using the “EdgeR” package, and the *P* values were adjusted for Bonferroni. The null model was used to quantitatively analyze the assembly mechanism of the phyllosphere microbial community of the two varieties under DBP exposure. The βNTI of bacteria community assembly was calculated by “picante” (77, 78). All data were checked for normality (Shapiro-Wilk test) and homogeneity of variances (Levene’s test). Statistical comparisons among treatments were analyzed by one-way ANOVA, followed by Tukey’s test. Statistical analyzes were considered significant at *P* < 0.05.

## Data Availability

All sequencing data have been uploaded to the NCBI database with the accession number PRJNA123864. All metabolites have been uploaded to the MetaboLights database with the accession number MTBLS12464.

## SUPPLEMENTAL MATERIAL

Supplemental material aem.00958-25-s0001.docx Texts S1 and S2, Fig. S1, and Tables S1 and S2.

ASM does not own the copyrights to Supplemental Material that may be linked to, or accessed through, an article. The authors have granted ASM a non-exclusive, world-wide license to publish the Supplemental Material files. Please contact the corresponding author directly for reuse.
