# Supplementary material for: Phyllosphere microbes are associated with variety-specific accumulation of di-n-butyl phthalate in choysum (Brassica parachinensis)
Source: Appl Environ Microbiol. 2025 Jul 22;91(8):e00958-25. doi: 10.1128/aem.00958-25 (PMC12366302; doi:10.1128/aem.00958-25)
Supplement: Supplemental material — Texts S1 and S2, Fig. S1, and Tables S1 and S2. [file aem.00958-25-s0001.docx]

**Supplementary Material**

**Phyllosphere microbes mediate variety-specific accumulation of di-n-butyl phthalate in choysum (*Brassica parachinensis*)**

Rui-Ting Wu^a,1^, Chang-Peng Ye^a,1^, Qiong Hu^a^, Yi-Min Gu^a^, Huan Du^b^, Miao-Yue Zhang^c^, Lei Xiang^a^, Bai-Lin Liu^a^, Yan-Wen Li^a^, Quan-Ying Cai^a^, Ce-Hui Mo^a,^*, Hai-Ming Zhao^a,c,^*

*^a^ Guangdong Provincial Research Center for Environment Pollution Control and Remediation Materials, College of Life Science and Technology, Jinan University, Guangzhou 510632, China*

*^b^ Guangzhou Customs Technology Center, No. 66 Huacheng Avenue, Tianhe District, Guangzhou 510623, China*

*^c^ Guangdong Provincial Key Laboratory of Environmental Pollution Control and Remediation Technology, Sun Yat-sen University, Guangzhou 510275, China*

*** Corresponding authors:**

Hai-Ming Zhao. Email: zhaohm99@jnu.edu.cn.

Ce-Hui Mo. Email: [tchmo@jnu.edu.cn](mailto:tchmo@jnu.edu.cn).

^1^These authors contribute equally to this paper.

# **Supplementary Texts**

## **Text S1.** **Soil preparation and experimental design**

The soil was air-dried and mixed thoroughly, and then sieved through a 2-mm stainless-steel sieve. The physiochemical properties of soil were identified as follows: 20.32 ± 1.22 g kg^-1^ of organic matter (dry weight, DW), 1.11 ± 0.01 g kg^-1^ of total N, 2.61 ± 0.06 g kg^-1^ of total P, 15.57 ± 0.25 g kg^-1^ of total K, 99 ± 0.4 cmol kg^-1^ of cation exchange capacity, and pH of 5.47± 0.08 (1: 2.5, w/v). No DBP was detected in the soil samples. According to the environmental concentrations identified in our previous investigation, soil spiked at 100 mg kg^-1^ of DBP was used as DBP treatment. An aliquot of soil (passed through a 2-mm sieve, 10% total quantity of soil) was spiked with DBP solution in acetone, after acetone evaporated, the spiked soils were mixed thoroughly with uncontaminated soils and sieved again to homogenize them. After the addition of 30% deionized water, the polluted soils were aged for two weeks in the dark to simulate actual contaminated soil. Following this aging period, the concentration of DBP in the simulated contaminated soil was measured to 76.73±6.87 mg/kg.

## **Text S2. Analysis of DBP**

Leaves samples were freeze-dried at -55°C (Thermo Heto PowerDry LL3000, USA) and ground to pass through a sieve (0.5 mm). Briefly, each 1.00 g (DW) of plant sample in 20-mL dichloromethane was extracted in an ultrasonic bath (Kedao SK2000LH, China) for 10 min three times, and then centrifuged at 3,500 rpm for 5 min. The supernatant was combined and concentrated in a vacuum rotary evaporator (Yarong RE-2000, China). The concentrated extract was loaded on a combined glass chromatography column (35-cm length × 1-cm i.d., pre-eluted with 20 mL of dichloromethane) of silica gel and anhydrous sodium sulfate, and eluted with 50 mL of dichloromethane three times. The eluate was concentrated successively using a vacuum rotary evaporator and under a gentle stream of nitrogen (Organomation N-EVAP112, USA), and adjusted to 1 mL with dichloromethane.

The extracts were analyzed using gas chromatography coupled with mass spectrometry (GC–MS, QP2010Plus, Shimadzu, Japan). A capillary column of HP-5 fused-silica (film thickness, 0.25 μm; inner diameter, 0.25 mm; length, 30 m) was used for separation. The GC temperature program of the oven was as follows: 100°C hold for 2 min, raised at 15°C min^-1^ to 129°C, then at 40°C min^-1^ to 280°C (hold for 5 min). The temperature of the injector and ion source was fixed at 250°C and 220°C, respectively. Helium (99.999% purity) was used as a carrier gas at a flow rate of 1.0 mL min^-1^. Sample extract (1.0 μL) was injected in splitless mode. The solvent delay was 1.00 min and total running time was 12.33 min. Mass spectra were acquired in the electron ionization (70 eV), and signal acquisition was performed in selected ion-monitoring (SIM) mode. The identity of the DBP peak was confirmed by comparison of the retention time of DBP standard and the characteristic ion (the primary characteristic ion of DBP being 149) in the mass spectra. Quantitative analysis was conducted using the calibration method based on a five-point calibration curve (0 to 4.0 mg L^-1^).

# **Supplementary Figure**

**Figure S1.** Alpha diversity (A), microbial assembly (B) and composition (C), differentially abundant phylum (D-F) in response to the two choysum varieties (LAV and HAV) under DBP exposure. Data are means ± SE (*n* = 6). The same lowercase letters represent no significant differences at the 0.05 level.

# **Supplementary Tables**

**Table S1.** Topological properties the phyllosphere microbial interaction network

| Group | Node | Edge | Average degree | Average clustering coefficient |
| --- | --- | --- | --- | --- |
| LAV_CK | 67 | 334 | 9.97 | 0.84 |
| LAV_DBP | 107 | 222 | 4.15 | 0.57 |
| HAV_CK | 102 | 578 | 11.33 | 0.70 |
| HAV_DBP | 41 | 196 | 9.56 | 0.73 |

**Table S2** Functional characterization of phyllosphere microorganisms

| Source | Strain | Nitrogen  fixation | Phosphate  solubilization(D/d) | Siderophore  (D/d) | DBP  degradation(%) |
| --- | --- | --- | --- | --- | --- |
| LAV | *B. altitudinis* | + | 1.54±0.14 | 1.76±0.15 | 78.6±6.0 |
| HAV | *S. yanoikuyae* | + | - | 2.12±0.49 | 81.9±6.4 |

Note: The '+' symbol denotes functional presence, whereas the '-' symbol indicates functional absence or detection below the threshold of significance.
